# Antibiotic resistance patterns of *Helicobacter pylori* strains isolated from the Tibet Autonomous Region, China

**DOI:** 10.1186/s12866-022-02613-y

**Published:** 2022-08-13

**Authors:** Xiaoqiong Tang, Zhonghua Wang, Yalin Shen, Xiaona Song, Mohammed Benghezal, Barry J. Marshall, Hong Tang, Hong Li

**Affiliations:** 1 West China Marshall Research Center for Infectious Diseases, Center of Infectious Diseases, West China Hospital, Sichuan University, Chengdu, China; 2 Division of Infectious Diseases, State Key Laboratory of Biotherapy and Center of Infectious Diseases, West China Hospital, Sichuan University, Chengdu, China; 3 Department of Gastroenterology, Tibet Autonomous Region People’s Hospital, Lhasa, China; 4 Helicobacter pylori Research Laboratory, School of Biomedical Sciences, Marshall Centre for Infectious Disease Research and Training, University of Western Australia, Nedlands, Australia; 5 School of Biomedical Engineering, Marshall Laboratory of Biomedical Engineering, Shenzhen University Health Science Center, Shenzhen, China

**Keywords:** *Helicobacter pylori*, Antibiotic resistance, Tibet Autonomous Region, China

## Abstract

**Background:**

The prevalence of *Helicobacter pylori* antibiotic susceptibility in the Tibet Autonomous Region, China is not determined. This study aimed to evaluate the antibiotic resistance patterns of *H. pylori* isolates there.

**Results:**

A total of 153 (38.5%) *H. pylori* strains were successfully isolated from 397 patients in People's Hospital of Tibet Autonomous Region, China. The overall resistance rates were as follows: clarithromycin (27.4%), levofloxacin (31.3%), metronidazole (86.2%), amoxicillin (15.6%), tetracycline (0%), furazolidone (0.6%), and rifampicin (73.2%). Only 2.0% of *H. pylori* isolates were susceptible to all tested antimicrobials, with mono resistance, dual resistance, triple resistance, quadruple resistance, and quintuple resistance being 18.3%, 44.4%, 18.3%, 12.4%, and 4.6%, respectively. The resistance rates to levofloxacin (40.5%) and amoxicillin (21.5%) in strains isolated from female patients were significantly higher than those from male patients (21.6% and 9.5%, respectively).

**Conclusions:**

This study demonstrates high *H. pylori* resistance rates to clarithromycin, levofloxacin, metronidazole, and rifampicin, whereas moderate resistance to amoxicillin, and negligible resistant to tetracycline, and furazolidone in Tibet Autonomous Region, China. The high resistance to rifampicin warns further investigation of its derivative, rifabutin.

**Supplementary Information:**

The online version contains supplementary material available at 10.1186/s12866-022-02613-y.

## Introduction

*Helicobacter pylori* (*H. pylori*) is a spiral and gram-negative pathogen that infects more than half of the world’s population, causing peptic ulcer, chronic gastritis, gastric atrophy, gastric intestinal metaplasia, gastric cancer and gastric mucosa-associated lymphoid tissue (MALT) lymphoma [1, 2]. Approximately 90% of non-cardia gastric cancer cases have been attributed to *H. pylori* infection. It was defined as a class I carcinogen by the World Health Organization (WHO) [3]. In the absence of an effective vaccine, the eradication of *H. pylori* through antibiotics has become the main strategy for resolving gastric lesions and preventing the occurrence of gastric cancer [2, 4, 5].

In recent years, the increasing resistance of *H. pylori* against commonly used antibiotics has posed a great challenge to its eradication [6]. For example, its resistance rate to clarithromycin (CLR) and metronidazole (MTZ) has generally risen to more than 15% worldwide, making the standard CLR or MTZ-based triple therapy no longer the first-line treatment [1]. To ensure an eradication rate exceeding 90%, treatment regimens based on antimicrobial susceptibility testing (AST) have been recommended [7, 8]. However, clinicians do not always have access to the resistance data for each *H. pylori* isolate, so the selection of therapeutic regimens based on the prevalence of antibiotic resistance remains the current important method.

Of note, the prevalence of *H. pylori* antibiotic resistance varies globally and even in a country [9, 10]. For instance, a meta-analysis demonstrated that the resistance rates to amoxicillin (AML) and tetracycline (TET) reached 38% and 13% in Africa, 14% and 10% in Eastern Mediterranean region, whereas the resistance rates to AML and TET in European region were nearly 0%. Therefore, region-specific surveillance of the *H. pylori* antibiotic resistance data has been recommended to guide appropriate choice of *H. pylori* eradication regimens [6].

To date, many studies have investigated the prevalence of *H. pylori* antibiotic resistance in northern, eastern, and southern China [11–15]. However, *H. pylori* antibiotic resistance data of the Tibet Autonomous Region, which is a high-altitude province located in West China, remains to be confirmed. In this study, we performed AST of *H. pylori* strains in this Tibetan region with seven antibiotics: CLR, levofloxacin (LEV), MTZ, AML, TET, rifampicin (RIF), furazolidone (FZD).

## Materials and methods

### Patients and *H. pylori* isolation

Patients from the Tibetan Autonomous Region scheduled for gastroscopy in the People's Hospital of Tibet Autonomous Region were enrolled between 2018 and 2020. Informed consent was obtained from all patients.

During gastroscopy, 1 or 2 biopsy specimens were taken from the gastric antrum and/or corpus and placed in sterile vials containing *H. pylori* preservation solution. The gastric specimens were then transferred in dry ice to the *H. pylori* laboratory at West China-Marshall Research Center for Infectious Diseases. The specimens were homogenized and incubated onto Columbia agar plates supplemented with 5% sheep blood, which were purchased from Autobio (China). After incubating the plates for 3–5 days at 37℃ under microaerophilic conditions (N_2_: H_2_: CO_2_, 85%: 5%: 10%), the colonies resembling *H. pylori* were expanded and confirmed by Gram-staining, oxidase, catalase and urease tests.

### Antimicrobial susceptibility testing

AST of the isolated *H. pylori* strains to CLR, LEV, MTZ, AML, TET and RIF was performed through an E-test (Liofilchem s.r.l, Italy), whereas the susceptibility to FZD was determined using a disk diffusion method as previously described [16]. In brief, the freshly grown strains were suspended in sterile saline, and the culture suspension was adjusted to a concentration of 1.0 OD_600_, or approximately 5 × 10^8^ CFU/mL. Subsequently, taken by a sterile cotton swab, 100 μL of the suspension was incubated on a Columbia blood agar plate. An E-test strip or a 6 mm-diameter disk containing 100 μg FZD (Oxoid, USA) was placed firmly on the plate. The plates were incubated at 37℃ under microaerophilic conditions for 3–5 days. Minimum inhibitory concentrations (MICs) were read by the intersection of the elliptical zone of growth inhibition with the MIC scale on the E-test strip, and inhibition zone diameters were measured in millimeters (mm) with a ruler according to the guidance of the European Committee on Antimicrobial Susceptibility Testing (EUCAST), version 10.0, 2020 [17].

Based on the recommendation of the EUCAST, resistance to CLR, LEV, MTZ, AML, TET and RIF was defined as MIC > 0.5 mg/L, MIC > 1 mg/L, MIC > 8 mg/L, MIC > 0.125 mg/L, MIC > 1 mg/L and MIC > 1 mg/L, respectively [17]. When the inhibition zone diameter was less than 21 mm, resistance to FZD was defined [18]. Strains exhibiting resistance to 3 or more of the tested antibiotics were defined as multi-drug resistant (MDR).

### Statistical analysis

All statistical analyses were performed with software SPSS version 16.0 (SPSS Inc., Chicago, USA). The chi-square test was used to assess the discrepancy in resistance rates in different gender, age and endoscopic finding groups. Statistical significance was regarded as *P* < 0.05.

## Results

From 2018 to 2020, 397 patients undergoing gastroscopy were enrolled in this study to have their gastric specimens cultured. A total of 157 *H. pylori* strains were isolated from these patients, giving a positive culturing rate of 38.5%. Since the contamination occurred during the culture of 4 *H. pylori* strains, finally, a total of 153 strains were tested for antimicrobial susceptibility. The baseline characteristics of these patients with susceptibility testing are shown in Table 1 and Table S1. Among them, the number of men and women was 74 and 79, respectively, and their mean age was 48.9 years. Most of these patients are Tibetans and come from Lhasa city. Among the patients with antibiotic susceptibility testing of *H. pylori* strains, chronic gastritis or duodenitis was found in 133 patients, and peptic ulcer was found in 45 patients.

**Table 1:** Characteristics of the 153 patients with antibiotic susceptibility testing of *H. pylori* isolates

Variables	Patients (*n* = 153)
Age (y)	48.9
Gender	
Male	74
Female	79
Ethnicity	
Tibetan nationality	150
Han nationality	2
Hui nationality	1
Region	
Lhasa	118
Chamdo	4
Nyingchi	8
Nakqu	10
Shigatse	5
Lhokha	3
Undetermined	5
Endoscopic findings	
Reflux esophagitis	16
Chronic Gastritis/duodenitis	133
Peptic ulcer	45
Gastric malignancy	2

### Individual antibiotic resistance rate

**Fig. 1:**
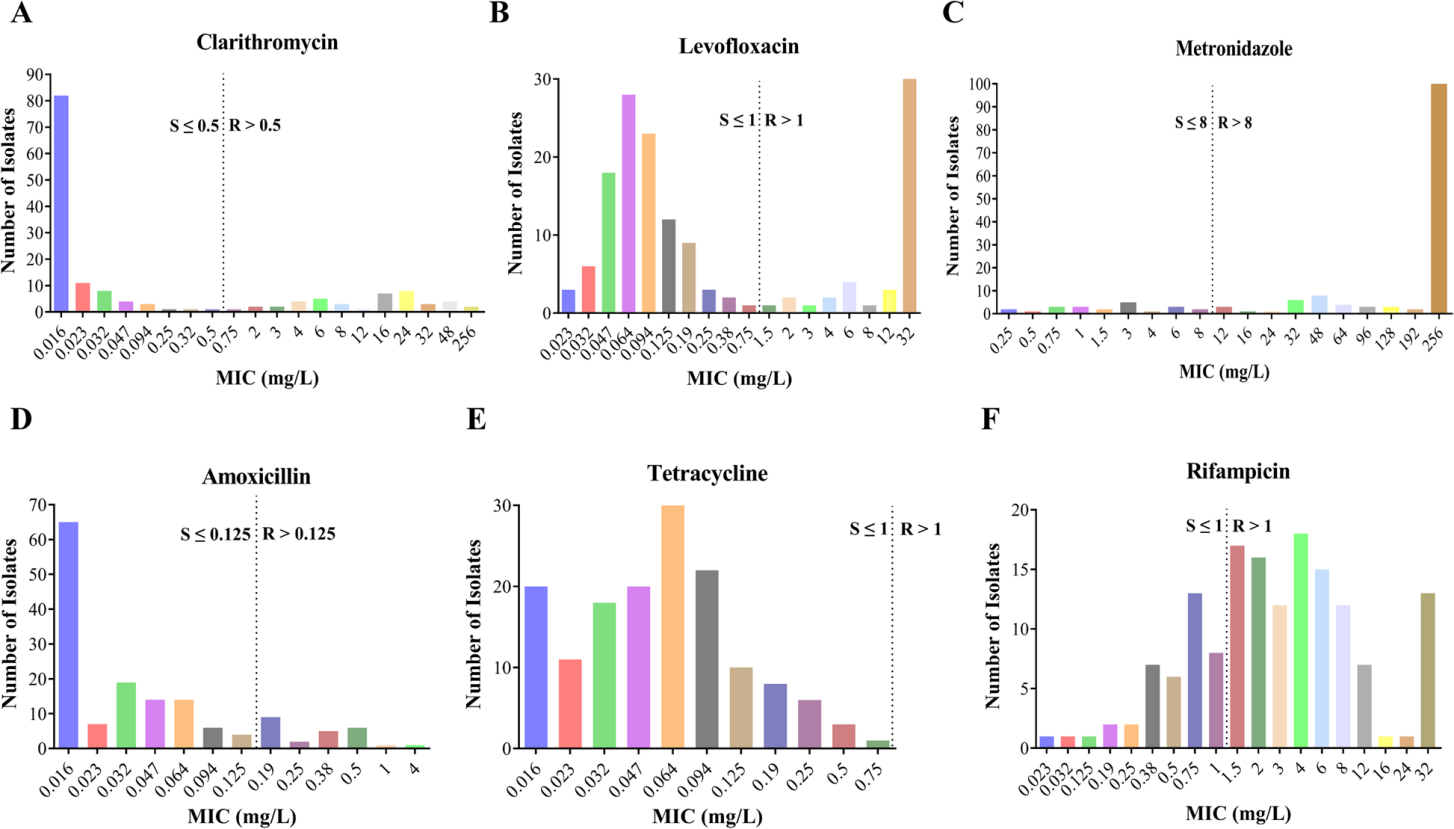
Minimum inhibitory concentrations (MICs) distribution of clarithromycin (**A**), levofloxacin (**B**), metronidazole (**C**), amoxicillin (**D**), tetracycline (**E**), and rifampicin (**F**) to 153 *H. pylori* isolates. Dashed arrows indicate the resistance breakpoints with MIC > 0.5 mg/L for clarithromycin, MIC > 1 mg/L for levofloxacin, MIC > 8 mg/L for metronidazole, MIC > 0.125 mg/L for amoxicillin, MIC > 1 mg/L for tetracycline and MIC > 1 mg/L for rifampicin. S, Sensitive; R, Resistant

To obtain a better understanding of antibiotic resistance in these *H. pylori* strains, we first counted the MIC distributions (Fig. 1, Table S1). For MTZ, almost two-thirds of the strains had MIC values of 256 mg/L, while the MICs of other one-thirds of the strains were uniformly distributed: 0.25–8 mg/L (*n* = 22) and 12–192 mg/L (*n* = 31). The MIC values against CLR, LEV and RIF were distributed widely. For the MIC values against CLR, 109 strains had MICs ≤ 0.25 mg/L, two strains had MICs of 0.32 and 0.5 mg/L, 19 strains had MICs in the range of 0.75–8 mg/L, and 25 strains had MICs from 12 to 256 mg/L. As for LEV, the strains had MICs of 0.023–0.75 mg/L (*n* = 106), 1.5–12 mg/L (*n* = 14), and 32 mg/L (*n* = 34). With regard to the MICs against RIF, the MIC values were more evenly distributed as follows: ≤ 1 mg/L (*n* = 41), 1.5–4 mg/L (*n* = 63) and 6–32 mg/L (*n* = 49) (Fig. 1).

Twenty-four strains were AML-resistant, among which, 11 strains had MICs of 0.19–0.25 mg/L, 11 had MICs of 0.38–0.5 mg/L, and the remaining 2 had MIC values of 1 mg/L and 4 mg/L. All 153 *H. pylori* strains were TET susceptible, with most of them having MIC values in the range of 0.016–0.125 mg/L. Using the 100 μg-FZD disk, 152 strains (99.4%) exhibited sensitivity to FZD, and 1 strain with inhibition zone diameter of 15 mm exhibited resistance to FZD (Fig. 1).

**Fig. 2:**
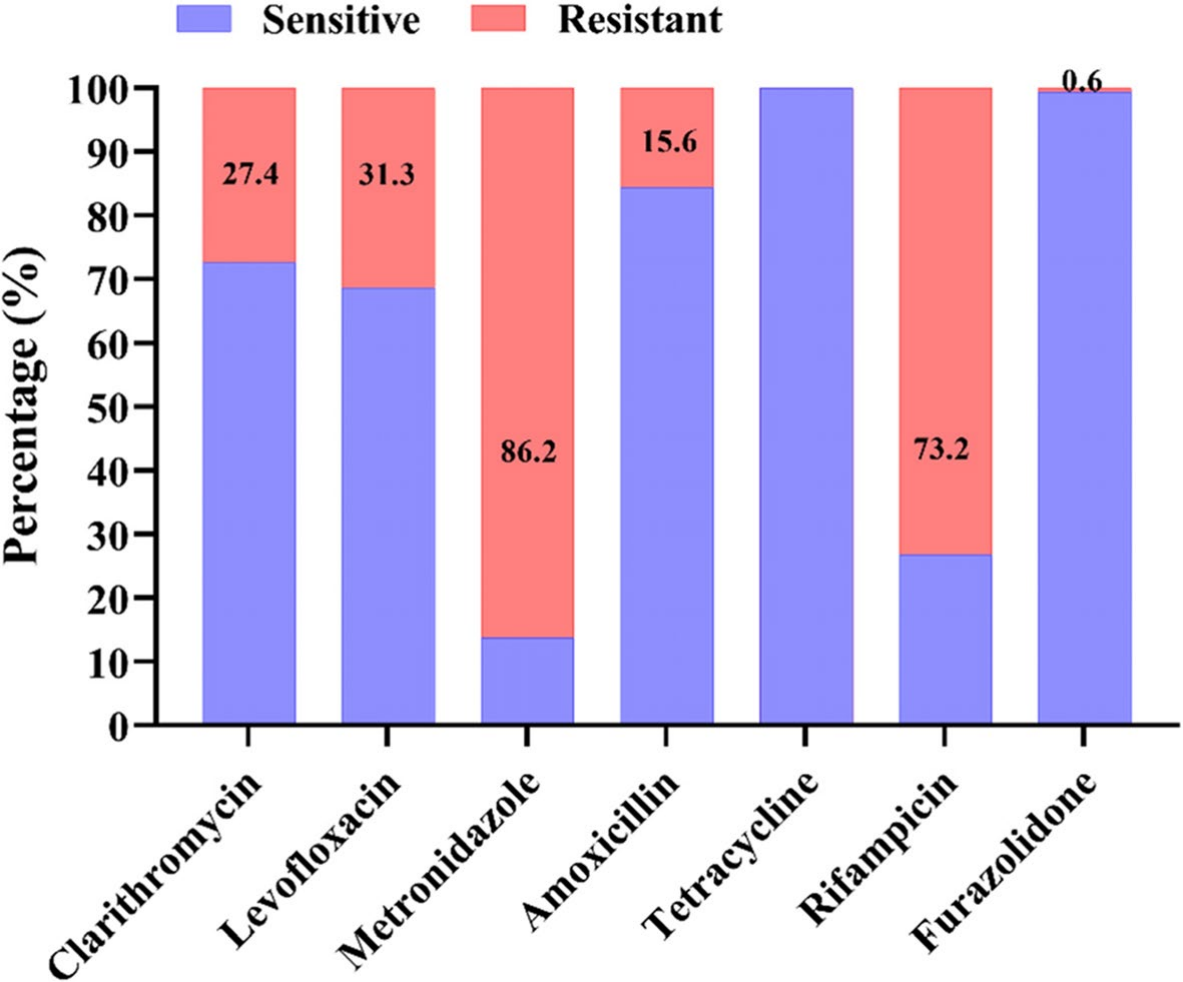
The overall sensitive and resistance rates of the 153 *H. pylori* isolates against seven antibiotics. The resistance to clarithromycin, levofloxacin, metronidazole, amoxicillin, tetracycline, rifampicin was determined by E-test, and the resistance to furazolidone was determined by disk diffusion method

Therefore, among the 153 strains tested for antibiotic susceptibility testing, the overall resistance rate to MTZ, CLR, LEV, RIF, AML, TET and FZD was 86.2%, 27.4%, 31.3%, 73.2%, 15.6%, 0.0% and 0.6%, respectively (Fig. 2).

### Multiple antibiotic resistance

Of the 153 *H. pylori* strains isolated from Tibet, only 3 (2.0%) were susceptible to all the seven tested antibiotics, 28 (18.3%) were resistant to one antibiotic (1 to CLR, 2 to LEV, 20 to MTZ, and 5 to RIF), and 68 (44.4%) exhibited dual resistance (including 51 to MTZ + RIF). MDR was observed in 54 (35.3%) strains, encompassing 28 (18.3%), 19 (12.4%), and 7 (4.6%) strains resistant to three, four and five antibiotics, respectively. Among the strains with triple and quadruple resistance, 10 were resistant to CLR + MTZ + RIF, and 9 were resistant to CLR + LEV + MTZ + RIF. All the seven strains showing quintuple resistance were resistant to CLR + LEV + MTZ + AML + RIF. Notably, simultaneous resistance to CLR and MTZ was found in 37 (24.1%) strains, including dual, triple, quadruple and quintuple resistant strains (Table 2).

**Table 2:** Resistance patterns among the 153 *H. pylori* isolates

Resistance patterns	n (%)
None resistance	3 (2.0)
Mono resistance	
CLR	1 (0.6)
LEV	2 (1.3)
MTZ	20 (13.1)
RIF	5 (3.3%)
Dual resistance	
CLR + RIF	3 (1.9%)
CLR + MTZ	1 (0.6%)
CLR + LEV	1 (0.6%)
LEV + MTZ	6 (3.9%)
LEV + RIF	4 (2.6%)
MTZ + RIF	51 (33.3%)
MTZ + FZD	1 (0.6%)
AML + RIF	1 (0.6%)
Triple resistance	
CLR + LEV + MTZ	4 (2.6%)
MTZ + AML + RIF	5 (3.3%)
LEV + MTZ + RIF	7 (4.6%)
CLR + MTZ + RIF	10 (6.5%)
CLR + MTZ + AML	1 (0.6%)
CLR + LEV + RIF	1 (0.6%)
Quadruple resistance	
CLR + LEV + MTZ + RIF	9 (5.9%)
CLR + LEV + MTZ + AML	1 (0.6%)
LEV + MTZ + AML + RIF	5 (3.3%)
CLR + MTZ + AML + RIF	3 (1.9%)
CLR + LEV + MTZ + AML	1 (0.6%)
Quintuple resistance	
CLR + LEV + MTZ + AML + RIF	7 (4.6%)

*CLR* Clarithromycin, *LEV* Levofloxacin, *MTZ* Metronidazole, *AML* Amoxicillin, *TET* Tetracycline, *RIF*, Rifampicin, *FZD* Furazolidone

### Factors influencing antibiotic resistance

By comparing the resistance rates of *H. pylori* in different genders, ages and endoscopic findings, we found that the resistance rate of *H. pylori* to LEV was 40.5% and to AML was 21.5% in the female group, which were much higher than those of 21.6% (*P* = 0.012) and 9.5% (*P* = 0.040), respectively, in the male group. In patients aged 18–40, 41–55, and ≥ 56 years, the resistance rates to CLR (17.6% vs. 27.4% vs. 35.7%), MTZ (82.3% vs. 85.2% vs. 92.8%), AML (11.7% vs. 15.7% vs. 17.8%), and RIF (70.5% vs. 72.2% vs. 78.5%) tended to increase, but there was no significant difference. Furthermore, discrepancies in resistance rates to antibiotics were also observed between patients with non-peptic ulcer and peptic ulcer diseases, but this was not statistically significant (Table 3). Thus, we can conclude that gender is the factor significantly associated with resistance to LEV and AML in *H. pylori* isolates from Tibet, China.

**Table 3:** Resistance comparisons of *H. pylori* isolates from patients with different genders, ages, and endoscopic findings

Antibiotic	Gender (n, %)		*P*	Age (n, %)			*P*	Endoscopic findings (n, %)		*P*
	Male (*n* = 74)	Female (*n* = 79)		18–40 (*n* = 17)	41–55 (*n* = 108)	≥ 56 (*n* = 28)		NPU (*n* = 108)	PU (*n* = 45)	
Clarithromycin	17 (22.9)	25 (31.6)	0.193	3 (17.6)	29 (27.4)	10 (35.7)	0.415	33 (31.1)	9 (20.0)	0.163
Levofloxacin	16 (21.6)	32 (40.5)	0.012 *	7 (41.1)	34 (31.5)	7 (25.0)	0.525	37 (34.3)	11 (24.4)	0.233
Metronidazole	62 (83.7)	70 (88.6)	0.386	14 (82.3)	92 (85.2)	26 (92.8)	0.508	95 (88.0)	37 (82.2)	0.347
Amoxicillin	7 (9.5)	17 (21.5)	0.040*	2 (11.7)	17 (15.7)	5 (17.8)	0.862	15 (13.9)	9 (20.0)	0.344
Rifampicin	54 (72.9)	58 (73.4)	0.951	12 (70.5)	78 (72.2)	22 (78.5)	0.770	76 (70.4)	36 (80.0)	0.220

*NPU* Non-peptic ulcer, *PU* Peptic ulcer

## Discussion

With the AST of 153 *H. pylori* strains isolated from patients in Tibet Autonomous Region, China, we found a very high resistance to MTZ (86.2%) and RIF (73.2%), a relatively high resistance to LEV (31.3%) and CLR (21.4%), a moderate resistance to AML (15.6%), and low resistance to TET (0.0%) and FZD (0.6%). In addition, the high prevalence of MDR strains should be highlighted (35.3% of strains were resistant to three, four, and five antibiotics). The results demonstrate a treatment challenge of *H. pylori* infection due to the high resistance to the commonly used antibiotics.

CLR and MTZ are two of the most commonly used antibiotics for respiratory tract or anaerobic infections. Frequent use of these antibiotics for these infections contributes to *H. pylori* resistance [19]. Although regionally variable, according to a meta-analysis of 178 studies, the resistance rates of *H. pylori* strains to them are increasing to more than 15% in nearly the whole world [10]. With regard to resistance in the northern, southeastern, and central regions of China, *H. pylori* resistance rates to CLR and MTZ were reported to be 19%-45% and 74–89%, respectively [12]. In our current study, the CLR resistance rate of 21.4% and MTZ of 86.2% in the Tibet Autonomous Region were within the range of those in other regions. Moreover, previous studies have shown that female patients carry higher CLR and MTZ resistance rates than male patients due to the higher incidence of treating gynecological diseases with the two antibiotics [20, 21]. Here, although the resistance rate between genders was not statistically significant, higher resistance to CLR and MTZ in female patients than in male patients was still observed, which was in line with the results of previous studies [20, 22]. As CLR resistance critically decreases the efficacy of standard CLR-based triple or quadruple therapy, the present result suggests the avoidance of CLR-containing regimens for *H. pylori* eradication without prior susceptibility testing in Tibet Autonomous Region, China. Although the eradication rate of *H. pylori* by MTZ-containing therapy can be improved by increasing its dosage, prolonging its duration or adding bismuth, the increased incidence of adverse side effects such as nausea, vomiting, swirling and rashes needs to be considered [23]. Therefore, it would be better to abandon MTZ-containing regimens as empirical treatment regimens for *H. pylori* eradication without prior susceptibility testing.

LEV-containing triple or quadruple regimen has been increasingly used for second-line eradication therapy [4]. A previous European multicenter study demonstrated a relatively low rate of LEV resistance of 14% [22]. Nevertheless, higher LEV resistance rates have been reported in Asia (up to 56% in China, 34% in Japan, and 28% in Korea) [6]. In this study, a high rate (31.1%) of LEV resistance was also observed, which surpasses the 25% threshold of resistance rate for choosing LEV as part of empirical or rescue regimens [24], discouraging LEV-containing regimens appropriate for *H. pylori* eradication without prior susceptibility testing. Of note, a higher rate (40.5%) of LEV resistance in female patients than that in male patients (21.5%) needs special attention, which may also be related to more consumption of LEV because of other respiratory tract, urinary tract or gynecological infectious diseases in female patients [20, 21].

In the current study, the rate of resistance to AML (15.6%) in the Tibet Autonomous Region, China was comparable to that reported in the Eastern Mediterranean region (14%) [10]. What should be noted is that in this study, AML resistance was defined when the MIC was more than the EUCAST recommended cut-off value of 0.125 mg/L. When we applied MIC > 1 mg/L as the AML resistance cut-off value used in a multi-region study [25], 23 strains with MICs of 0.19, 0.25, 0.5 and 1 mg/L were redefined as susceptible, giving an AML resistance rate down to 0.6% in the Tibet region, comparable to the overall AML resistance rate of 3.4% in China [25]. Hence, the AML resistance role is negligible in clinical practice, which means that this antibiotic could be prescribed in most cases.

Similar to AML, the TET and FZD resistance rates were also low at 0% and 0.6%, respectively, which were in accordance with the resistance rates reported in other regions of China [9, 13]. Regimens including these two antimicrobials without prior susceptibility testing are expected to achieve high eradication efficacy in patients there. Unfortunately, they are not generally available in China, affecting their clinical application. Semisynthetic TET derivatives, including minocycline, are easily obtained in the clinic, and their antibacterial activity is higher than that of TET. It is interesting to test the sensitivity of *H. pylori* to the TET derivatives to determine whether they can be used as alternatives of TET for treating *H. pylori* infection.

Rifamycins, including RIF, rifabutin, rifaximin and rifapentine, are transcriptional inhibitor antibiotics that suppress bacterial DNA-directed RNA polymerase [26]. Among them, rifabutin plays an important role in salvage treatment of *H. pylori* infection [5, 27]. Previous studies demonstrated that the resistance rate in *H. pylori* to RIF was 1.0%, with full agreement with the rifabutin resistance rate (1.0%) when the breakpoint of RIF resistance was > 4 mg/L [26]. Therefore, they considered that there is cross-resistance between RIF and rifabutin in *H. pylori* and suggested RIF susceptibility testing to be used for screening rifabutin resistance [26]. In this study, we found that the resistance rate of *H. pylori* against RIF was much higher (73.2%) than the previously reported rate of 1.0% [28, 29]. Even though the breakpoint of RIF resistance > 4 mg/L was used as suggested by Hays et al. [26], resistance to RIF was still high at 32.0%. The higher RIF resistance rate might be because of two reasons. 1) In many previous articles, resistance to RIF was still detected using the disk-diffusion method (despite the MIC value provided by EUCAST) [30, 31], however, E-test was used to detect RIF resistance in our study. Hence the frequency of resistance may be different. 2) The wide use of RIF for treating tuberculosis, a disease with high prevalence in Tibet Autonomous Region, China, leads to a higher resistance rate of *H. pylori* to RIF [32]. Of note, if cross-resistance between rifabutin and RIF truly exists, the high RIF resistance rate in *H. pylori* strains in Tibet, China, would indicate the severity of rifabutin resistance. Nevertheless, it is worth noting that the rifabutin MICs of RIF-resistant strains were not known in previous studies [26, 33], and cross-resistance between RIF and rifabutin needs to be further confirmed in RIF-resistant strains isolated from the Tibetan region of China.

The comprehensive information on *H. pylori* resistance to 7 antibiotics in this study will be very helpful to select the optimal eradication regimens in the Tibet Autonomous Region, China. Nevertheless, two limitations of our study should be noted. First, we did not know whether the patients had undergone eradication therapy prior to this study. Therefore, the primary or secondary *H. pylori* resistance rate was not confirmed. Second, our findings were based on adult patients in a single hospital. Most of the included patients were from Lhasa city and only few were from other parts of Tibet, so this study may not reflect the general resistance of *H. pylori* strains in the whole population of the Tibetan region. Future studies are needed to include more patients (with the inclusion of less than 18 years old and from more hospitals) to determine the general antibiotic resistance in *H. pylori* strains from Tibet Autonomous Region, China.

## Conclusions

In conclusion, the high resistance to CLR, MTZ and LEV makes them unsuitable for empirical eradication of *H. pylori* in the Tibet Autonomous Region, China, only if susceptibility testing is performed to confirm the lack of resistance to these antibiotics before treatment. The moderate resistance to AML and negligible resistance to TET and FZD suggest that treatment regimens including two of these antimicrobials without prior susceptibility testing are expected to achieve high eradication efficacy in patients there.

## Supplementary Information

**Additional file 1:** **Table S1.** Patients’ characteristics and antibiotic susceptibility testing results for the 153 *H. pylori* strains.

## Data Availability

The datasets supporting the results and conclusion of this study are included within the article and the supplementary file.

## Abbreviations

MALT: Mucosa-associated lymphoid tissue
AST: Antimicrobial susceptibility testing
CLR: Clarithromycin
MTZ: Metronidazole
LEV: Levofloxacin
AML: Amoxicillin
TET: Tetracycline
FZD: Furazolidone
RIF: Rifampicin
MICs: Minimum inhibitory concentrations
MDR: Multi-drug resistant
