# Supplementary material for: Antibiotic resistance patterns of Helicobacter pylori strains isolated from the Tibet Autonomous Region, China
Source: BMC Microbiol. 2022 Aug 13;22:196. doi: 10.1186/s12866-022-02613-y (PMC9375320; doi:10.1186/s12866-022-02613-y)
Supplement: Supplementary file 1 — Additional file 1: Table S1. Patients’ characteristics and antibiotic susceptibility testing results for the 153 H. pylori strains. [file 12866_2022_2613_MOESM1_ESM.docx]

**Table S1.** Patients’ characteristics and antibiotic susceptibility testing results for the 153 *H. pylori* strains

|  |  |  |  |  |  | Minimum inhibitory concentrations (mg/L) | | | | | | | | Inhibition zone  diameter (mm) | | |  |
| --- | --- | --- | --- | --- | --- | --- | --- | --- | --- | --- | --- | --- | --- | --- | --- | --- | --- |
| No. | Gender | Age (y) | Nationality | Region | Endoscopic findings | CLR | LEV | MTZ | AML |  | TET | | RIF | | FZD |  |  |
| 1 | Male | 52 | Lhasa | Lhasa | GU+CD | 0.094 | 0.064 | 0.75 | 0.016 | 0.032 | | 2 | | 51 | | |  |
| 2 | Female | 36 | Nyingchi | Nyingchi | CG | 4 | 32 | 256 | 0.016 | 0.016 | | 0.38 | | 75 | | |  |
| 3 | Male | 27 | Nakqu | Nakqu | CG+GU | 0.023 | 0.047 | 256 | 0.25 | 0.032 | | 1.5 | | 61 | | |  |
| 4 | Male | 42 | Lhasa | Lhasa | GU | 0.016 | 32 | 256 | 0.032 | 0.094 | | 0.5 | | 48 | | |  |
| 5 | Male | 27 | Chamdo | Chamdo | CG | 0.023 | 32 | 6 | 0.016 | 0.047 | | 6 | | 67 | | |  |
| 6 | Male | 30 | Lhasa | Lhasa | GU | 0.016 | 0.032 | 256 | 0.016 | 0.064 | | 2 | | 74 | | |  |
| 7 | Female | 27 | Lhasa | Lhasa | CG | 0.094 | 0.047 | 256 | 0.016 | 0.023 | | 6 | | 50 | | |  |
| 8 | Female | 51 | Lhasa | Lhasa | CG | 24 | 32 | 256 | 0.016 | 0.25 | | 0.75 | | 65 | | |  |
| 9 | Female | 40 | Lhokha | Lhokha | CG | 0.016 | 0.023 | 256 | 0.023 | 0.19 | | 12 | | 54 | | |  |
| 10 | Female | 46 | Lhasa | Lhasa | CG | 0.016 | 0.047 | 256 | 0.094 | 0.023 | | 1 | | 70 | | |  |
| 11 | Male | 40 | Lhasa | Lhasa | CG | 0.016 | 0.032 | 256 | 0.064 | 0.047 | | 1.5 | | 70 | | |  |
| 12 | Male | 51 | Lhasa | Lhasa | RE+CG | 2 | 1.5 | 256 | 0.016 | 0.032 | | 1.5 | | 78 | | |  |
| 13 | Male | 53 | Lhasa | Lhasa | CG | 0.016 | 6 | 256 | 0.016 | 0.016 | | 32 | | 73 | | |  |
| 14 | Female | 52 | Lhasa | Lhasa | CG | 0.016 | 0.032 | 8 | 0.016 | 0.016 | | 1 | | 78 | | |  |
| 15 | Female | 50 | Lhasa | Lhasa | CG | 0.25 | 32 | 256 | 0.125 | 0.064 | | 1.5 | | 78 | | |  |
| 16 | Female | 59 | Lhasa | Lhasa | CG | 0.023 | 0.094 | 256 | 0.064 | 0.023 | | 0.032 | | 58 | | |  |
| 17 | Female | 51 | Lhasa | Lhasa | CG | 0.016 | 32 | 6 | 0.016 | 0.032 | | 1.5 | | 70 | | |  |
| 18 | Male | 54 | Nakqu | Nakqu | CG | 16 | 6 | 48 | 0.38 | 0.023 | | 0.75 | | 62 | | |  |
| 19 | Male | 54 | Lhokha | Lhokha | CG | 24 | 6 | 256 | 0.032 | 0.064 | | 1.5 | | 50 | | |  |
| 20 | Female | 40 | Shigatse | Shigatse | RE+CG | 6 | 8 | 256 | 0.023 | 0.032 | | 4 | | 45 | | |  |
| 21 | Male | 55 | Chamdo | Chamdo | RE+DU | 0.023 | 0.125 | 32 | 0.016 | 0.032 | | 12 | | 60 | | |  |
| 22 | Female | 46 | Nakqu | Nakqu | CG+GU | 16 | 0.047 | 256 | 0.064 | 0.047 | | 8 | | 65 | | |  |
| 23 | Male | 51 | Lhasa | Lhasa | RE+CG+DU | 0.016 | 32 | 1 | 0.016 | 0.064 | | 32 | | 80 | | |  |
| 24 | Male | 48 | Shigatse | Shigatse | CG | 8 | 0.125 | 256 | 0.125 | 0.094 | | 3 | | 73 | | |  |
| 25 | Male | 41 | Lhasa | Lhasa | GU | 0.016 | 0.064 | 32 | 0.016 | 0.064 | | 0.75 | | 71 | | |  |
| 26 | Female | 42 | Lhasa | Lhasa | CG | 4 | 32 | 256 | 0.19 | 0.064 | | 2 | | 57 | | |  |
| 27 | Female | 41 | Lhasa | Lhasa | CG | 0.016 | 0.064 | 256 | 0.016 | 0.094 | | 2 | | 65 | | |  |
| 28 | Male | 47 | Lhasa | Lhasa | GU | 0.016 | 0.064 | 256 | 0.047 | 0.064 | | 0.75 | | 75 | | |  |
| 29 | Female | 40 | Lhasa | Lhasa | CG | 0.016 | 32 | 256 | 0.047 | 0.047 | | 6 | | 60 | | |  |
| 30 | Male | 54 | Lhasa | Lhasa | DU | 0.016 | 0.19 | 256 | 0.064 | 0.25 | | 32 | | 53 | | |  |
| 31 | Female | 61 | Lhasa | Lhasa | CG | 0.016 | 0.094 | 64 | 0.016 | 0.047 | | 3 | | 70 | | |  |
| 32 | Female | 41 | Lhasa | Lhasa | CG+DU | 16 | 32 | 256 | 0.19 | 0.016 | | 3 | | 75 | | |  |
| 33 | Male | 61 | Lhasa | Lhasa | RE+DU | 0.016 | 0.047 | 3 | 0.016 | 0.032 | | 0.75 | | 80 | | |  |
| 34 | Female | 55 | Shigatse | Shigatse | CG | 0.016 | 32 | 0.5 | 0.064 | 0.094 | | 32 | | 80 | | |  |
| 35 | Male | 44 | Lhokha | Lhokha | CG | 0.016 | 32 | 64 | 0.016 | 0.016 | | 4 | | 80 | | |  |
| 36 | Male | 45 | Lhasa | Lhasa | CG | 0.016 | 0.38 | 256 | 0.016 | 0.064 | | 8 | | 80 | | |  |
| 37 | Male | 61 | Lhasa | Lhasa | CG | 3 | 0.047 | 256 | 0.016 | 0.094 | | 1.5 | | 75 | | |  |
| 38 | Female | 43 | Lhasa | Lhasa | CG | 6 | 32 | 256 | 0.023 | 0.016 | | 32 | | 85 | | |  |
| 39 | Female | 51 | Lhasa | Lhasa | CG | 0.016 | 0.064 | 192 | 0.016 | 0.016 | | 0.25 | | 85 | | |  |
| 40 | Female | 39 | Lhasa | Lhasa | CG | 0.016 | 32 | 256 | 0.5 | 0.125 | | 12 | | 39 | | |  |
| 41 | Female | 41 | Chamdo | Chamdo | CG | 0.016 | 0.094 | 12 | 0.032 | 0.064 | | 32 | | 65 | | |  |
| 42 | Male | 45 | Lhasa | Lhasa | CG | 0.016 | 0.047 | 3 | 0.016 | 0.064 | | 12 | | 68 | | |  |
| 43 | Male | 44 | Lhasa | Lhasa | CG+DU | 0.016 | 0.064 | 256 | 0.016 | 0.023 | | 1.5 | | 85 | | |  |
| 44 | Male | 44 | Lhasa | Lhasa | CG | 0.016 | 0.064 | 256 | 0.047 | 0.064 | | 0.023 | | 53 | | |  |
| 45 | Male | 44 | Lhasa | Lhasa | CG+GU | 0.016 | 32 | 96 | 0.19 | 0.047 | | 2 | | 85 | | |  |
| 46 | Female | 43 | Lhasa | Lhasa | CG | 0.023 | 0.25 | 256 | 0.125 | 0.25 | | 8 | | 39 | | |  |
| 47 | Male | 49 | Undetermined | Undetermined | CG | 0.016 | 32 | 24 | 0.5 | 0.094 | | 2 | | 52 | | |  |
| 48 | Female | 65 | Nyingchi | Nyingchi | CG | 0.023 | 0.25 | 256 | 0.38 | 0.5 | | 3 | | 36 | | |  |
| 49 | Female | 45 | Lhasa | Lhasa | CG | 0.016 | 0.19 | 256 | 0.38 | 0.25 | | 1.5 | | 38 | | |  |
| 50 | Female | 57 | Lhasa | Lhasa | CG | 0.016 | 0.125 | 128 | 0.094 | 0.094 | | 4 | | 45 | | |  |
| 51 | Female | 55 | Lhasa | Lhasa | CG | 0.016 | 0.047 | 16 | 0.047 | 0.016 | | 8 | | 75 | | |  |
| 52 | Male | 61 | Lhasa | Lhasa | RE+DU | 0.023 | 0.094 | 256 | 0.5 | 0.047 | | 12 | | 65 | | |  |
| 53 | Female | 41 | Undetermined | Undetermined | CG+DU | 2 | 0.094 | 256 | 0.125 | 0.032 | | 1.5 | | 55 | | |  |
| 54 | Female | 42 | Nyingchi | Nyingchi | CG | 0.016 | 0.064 | 256 | 0.016 | 0.016 | | 4 | | 80 | | |  |
| 55 | Male | 41 | Lhasa | Lhasa | CG | 0.023 | 0.125 | 256 | 0.016 | 0.094 | | 3 | | 50 | | |  |
| 56 | Male | 48 | Nakqu | Nakqu | CG | 24 | 0.38 | 0.75 | 0.094 | 0.016 | | 0.38 | | 85 | | |  |
| 57 | Female | 55 | Shigatse | Shigatse | GU | 16 | 32 | 256 | 0.38 | 0.125 | | 8 | | 72 | | |  |
| 58 | Male | 46 | Nakqu | Nakqu | CG+GU | 0.016 | 0.094 | 256 | 0.023 | 0.125 | | 1 | | 70 | | |  |
| 59 | Male | 44 | Lhasa | Lhasa | CG | 0.016 | 0.047 | 256 | 0.016 | 0.125 | | 8 | | 62 | | |  |
| 60 | Female | 50 | Lhasa | Lhasa | CG | 48 | 0.125 | 256 | 0.38 | 0.19 | | 32 | | 44 | | |  |
| 61 | Female | 55 | Lhasa | Lhasa | CG | 0.016 | 12 | 256 | 0.016 | 0.064 | | 0.38 | | 44 | | |  |
| 62 | Male | 53 | Lhasa | Lhasa | CG | 0.016 | 0.094 | 256 | 0.016 | 0.047 | | 1 | | 15 | | |  |
| 63 | Male | 60 | Lhasa | Lhasa | RE+CG+GU+DU | 0.016 | 0.032 | 12 | 0.016 | 0.016 | | 8 | | 72 | | |  |
| 64 | Male | 58 | Lhasa | Lhasa | RE+CG+GU | 0.016 | 0.047 | 12 | 0.047 | 0.016 | | 32 | | 80 | | |  |
| 65 | Male | 40 | Undetermined | Undetermined | CG | 48 | 0.023 | 3 | 0.016 | 0.016 | | 8 | | 70 | | |  |
| 66 | Male | 47 | Undetermined | Undetermined | CG+DU | 0.016 | 0.094 | 256 | 0.016 | 0.023 | | 2 | | 75 | | |  |
| 67 | Male | 54 | Lhasa | Lhasa | CG | 0.032 | 0.064 | 48 | 0.064 | 0.023 | | 6 | | 68 | | |  |
| 68 | Male | 55 | Nakqu | Nakqu | GU | 0.023 | 0.064 | 256 | 0.016 | 0.064 | | 6 | | 58 | | |  |
| 69 | Male | 48 | Lhasa | Lhasa | CG | 0.032 | 0.19 | 256 | 0.094 | 0.125 | | 4 | | 58 | | |  |
| 70 | Female | 45 | Lhasa | Lhasa | GU | 0.016 | 0.094 | 6 | 0.023 | 0.032 | | 4 | | 52 | | |  |
| 71 | Female | 59 | Lhasa | Lhasa | CG | 0.016 | 0.19 | 256 | 0.047 | 0.064 | | 8 | | 45 | | |  |
| 72 | Male | 55 | Lhasa | Lhasa | RE+DU | 0.016 | 2 | 256 | 0.016 | 0.023 | | 32 | | 66 | | |  |
| 73 | Male | 51 | Lhasa | Lhasa | CG+DU | 0.032 | 0.064 | 256 | 0.016 | 0.032 | | 1.5 | | 56 | | |  |
| 74 | Male | 41 | Lhasa | Lhasa | CG | 0.016 | 0.047 | 256 | 0.032 | 0.023 | | 1.5 | | 70 | | |  |
| 75 | Male | 55 | Lhasa | Lhasa | RE+CG | 0.016 | 0.064 | 32 | 0.016 | 0.016 | | 24 | | 78 | | |  |
| 76 | Male | 42 | Lhasa | Lhasa | CG+DU | 0.016 | 0.094 | 256 | 0.064 | 0.032 | | 1.5 | | 60 | | |  |
| 77 | Male | 46 | Lhasa | Lhasa | RE+CG | 3 | 0.047 | 256 | 0.016 | 0.064 | | 0.38 | | 70 | | |  |
| 78 | Male | 62 | Lhasa | Lhasa | CG | 0.016 | 0.064 | 256 | 0.023 | 0.094 | | 1 | | 70 | | |  |
| 79 | Female | 42 | Lhasa | Lhasa | CG+DU | 0.032 | 2 | 256 | 0.19 | 0.125 | | 32 | | 59 | | |  |
| 80 | Female | 56 | Lhasa | Lhasa | Ca | 0.016 | 3 | 1 | 0.016 | 0.094 | | 0.19 | | 75 | | |  |
| 81 | Female | 65 | Lhasa | Lhasa | CG | 24 | 32 | 256 | 1 | 0.094 | | 0.75 | | 50 | | |  |
| 82 | Male | 60 | Lhasa | Lhasa | CG+DU | 0.016 | 0.094 | 256 | 0.016 | 0.094 | | 0.75 | | 70 | | |  |
| 83 | Female | 61 | Lhasa | Lhasa | CG | 48 | 32 | 256 | 0.19 | 0.75 | | 4 | | 44 | | |  |
| 84 | Female | 59 | Lhasa | Lhasa | CG+DU | 32 | 0.75 | 128 | 0.047 | 0.064 | | 3 | | 60 | | |  |
| 85 | Male | 51 | Nakqu | Nakqu | RE+CG+GU | 0.047 | 0.19 | 256 | 0.064 | 0.125 | | 32 | | 33 | | |  |
| 86 | Female | 52 | Lhasa | Lhasa | CG | 8 | 32 | 256 | 0.016 | 0.25 | | 4 | | 52 | | |  |
| 87 | Female | 78 | Nakqu | Nakqu | CG | 12 | 32 | 256 | 0.016 | 0.19 | | 6 | | 40 | | |  |
| 88 | Female | 55 | Nyingchi | Nyingchi | CG | 0.016 | 0.064 | 64 | 0.064 | 0.064 | | 4 | | 60 | | |  |
| 89 | Female | 47 | Nyingchi | Nyingchi | CG | 0.016 | 0.047 | 32 | 0.016 | 0.016 | | 0.38 | | 62 | | |  |
| 90 | Male | 54 | Lhasa | Lhasa | CG | 0.016 | 0.047 | 48 | 0.016 | 0.016 | | 0.25 | | 80 | | |  |
| 91 | Male | 46 | Lhasa | Lhasa | CG | 0.023 | 0.064 | 48 | 0.032 | 0.094 | | 16 | | 65 | | |  |
| 92 | Male | 50 | Lhasa | Lhasa | CG | 6 | 0.064 | 0.75 | 0.023 | 0.047 | | 6 | | 85 | | |  |
| 93 | Male | 55 | Lhasa | Lhasa | CG | 0.047 | 0.064 | 96 | 0.016 | 0.032 | | 8 | | 62 | | |  |
| 94 | Female | 49 | Lhasa | Lhasa | CG | 4 | 0.094 | 256 | 0.19 | 0.064 | | 3 | | 65 | | |  |
| 95 | Male | 53 | Lhasa | Lhasa | CG | 32 | 4 | 256 | 0.19 | 0.094 | | 6 | | 55 | | |  |
| 96 | Female | 50 | Lhasa | Lhasa | CG | 0.016 | 0.064 | 256 | 0.032 | 0.064 | | 4 | | 63 | | |  |
| 97 | Female | 59 | Nyingchi | Nyingchi | CG | 0.016 | 0.064 | 256 | 0.032 | 0.016 | | 6 | | 62 | | |  |
| 98 | Male | 61 | Nyingchi | Nyingchi | CG | 24 | 0.064 | 256 | 0.032 | 0.064 | | 3 | | 56 | | |  |
| 99 | Male | 66 | Chamdo | Chamdo | CG | 0.016 | 0.094 | 256 | 0.047 | 0.25 | | 1.5 | | 53 | | |  |
| 100 | Male | 56 | Nyingchi | Nyingchi | GU | 8 | 0.064 | 256 | 0.016 | 0.094 | | 12 | | 57 | | |  |
| 101 | Male | 66 | Lhasa | Lhasa | RE+GU | 24 | 12 | 256 | 0.016 | 0.047 | | 1.5 | | 65 | | |  |
| 102 | Female | 55 | Lhasa | Lhasa | CG | 0.016 | 32 | 256 | 0.032 | 0.064 | | 3 | | 66 | | |  |
| 103 | Male | 65 | Lhasa | Lhasa | CG | 0.016 | 32 | 256 | 0.032 | 0.19 | | 4 | | 66 | | |  |
| 104 | Male | 45 | Lhasa | Lhasa | CG+CD | 0.016 | 0.064 | 256 | 0.016 | 0.047 | | 2 | | 70 | | |  |
| 105 | Female | 40 | Lhasa | Lhasa | CG | 0.023 | 12 | 256 | 0.094 | 0.064 | | 0.5 | | 56 | | |  |
| 106 | Female | 58 | Nakqu | Nakqu | CG | 0.016 | 0.094 | 256 | 0.032 | 0.064 | | 4 | | 50 | | |  |
| 107 | Female | 48 | Lhasa | Lhasa | CG | 0.016 | 0.094 | 48 | 0.016 | 0.094 | | 2 | | 65 | | |  |
| 108 | Female | 40 | Lhasa | Lhasa | RE+CG | 0.016 | 0.125 | 256 | 0.016 | 0.032 | | 0.38 | | 65 | | |  |
| 109 | Male | 42 | Lhasa | Lhasa | CG | 0.016 | 0.094 | 256 | 0.016 | 0.047 | | 1 | | 60 | | |  |
| 110 | Female | 42 | Lhasa | Lhasa | CG | 0.016 | 0.047 | 0.25 | 0.016 | 0.094 | | 0.125 | | 65 | | |  |
| 111 | Female | 47 | Lhasa | Lhasa | CG+DU | 0.016 | 0.064 | 256 | 0.047 | 0.032 | | 32 | | 65 | | |  |
| 112 | Female | 24 | Lhasa | Lhasa | CG | 0.032 | 0.19 | 256 | 0.064 | 0.094 | | 1 | | 63 | | |  |
| 113 | Male | 51 | Lhasa | Lhasa | Ca | 0.016 | 0.064 | 1.5 | 0.016 | 0.047 | | 0.75 | | 75 | | |  |
| 114 | Female | 42 | Nakqu | Nakqu | CG | 24 | 32 | 256 | 0.047 | 0.032 | | 4 | | 76 | | |  |
| 115 | Female | 40 | Lhasa | Lhasa | CG | 0.016 | 32 | 256 | 0.032 | 0.032 | | 0.75 | | 55 | | |  |
| 116 | Male | 47 | Lhasa | Lhasa | CG | 0.016 | 0.094 | 256 | 0.032 | 0.125 | | 2 | | 72 | | |  |
| 117 | Female | 58 | Lhasa | Lhasa | CG | 0.032 | 0.125 | 256 | 0.016 | 0.19 | | 3 | | 57 | | |  |
| 118 | Female | 56 | Lhasa | Lhasa | CG | 32 | 32 | 256 | 0.5 | 0.5 | | 2 | | 38 | | |  |
| 119 | Male | 49 | Lhasa | Lhasa | CG | 0.016 | 0.125 | 256 | 0.032 | 0.047 | | 8 | | 76 | | |  |
| 120 | Female | 51 | Lhasa | Lhasa | CG | 0.016 | 4 | 0.25 | 0.016 | 0.047 | | 0.38 | | 85 | | |  |
| 121 | Male | 52 | Lhasa | Lhasa | CG | 0.032 | 0.125 | 256 | 0.016 | 0.064 | | 12 | | 77 | | |  |
| 122 | Female | 42 | Lhasa | Lhasa | CG | 0.5 | 0.094 | 256 | 0.016 | 0.047 | | 2 | | 60 | | |  |
| 123 | Female | 54 | Shigatse | Shigatse | GU | 0.016 | 32 | 256 | 0.19 | 0.064 | | 32 | | 58 | | |  |
| 124 | Male | 46 | Lhasa | Lhasa | CG+GU | 16 | 32 | 1.5 | 0.047 | 0.023 | | 0.5 | | 85 | | |  |
| 125 | Male | 52 | Lhasa | Lhasa | CG | 0.016 | 6 | 192 | 0.016 | 0.19 | | 1 | | 80 | | |  |
| 126 | Female | 50 | Lhasa | Lhasa | CG | 16 | 0.094 | 256 | 0.032 | 0.023 | | 2 | | 75 | | |  |
| 127 | Male | 47 | Lhasa | Lhasa | CG+DU | 0.094 | 0.125 | 256 | 0.032 | 0.064 | | 6 | | 45 | | |  |
| 128 | Female | 46 | Lhasa | Lhasa | CG | 0.75 | 32 | 256 | 0.032 | 0.094 | | 4 | | 72 | | |  |
| 129 | Male | 50 | Lhasa | Lhasa | RE+CG | 16 | 0.064 | 256 | 4 | 0.125 | | 4 | | 86 | | |  |
| 130 | Female | 48 | Lhasa | Lhasa | CG+DU | 0.016 | 0.047 | 256 | 0.016 | 0.016 | | 3 | | 75 | | |  |
| 131 | Male | 52 | Lhasa | Lhasa | RE+CG | 0.032 | 0.064 | 48 | 0.016 | 0.016 | | 0.5 | | 75 | | |  |
| 132 | Female | 46 | Lhasa | Lhasa | CG | 0.32 | 32 | 256 | 0.047 | 0.064 | | 4 | | 58 | | |  |
| 133 | Male | 41 | Lhasa | Lhasa | CG+DU | 0.016 | 0.19 | 48 | 0.016 | 0.19 | | 6 | | 59 | | |  |
| 134 | Male | 50 | Lhasa | Lhasa | CG | 0.016 | 0.19 | 128 | 0.064 | 0.094 | | 0.75 | | 67 | | |  |
| 135 | Female | 45 | Lhasa | Lhasa | CG | 6 | 32 | 256 | 0.032 | 0.064 | | 0.75 | | 65 | | |  |
| 136 | Female | 44 | Lhasa | Lhasa | CG | 0.016 | 0.047 | 32 | 0.016 | 0.032 | | 2 | | 85 | | |  |
| 137 | Female | 54 | Lhasa | Lhasa | CG | 0.047 | 0.25 | 256 | 0.25 | 0.5 | | 8 | | 55 | | |  |
| 138 | Female | 43 | Lhasa | Lhasa | GU | 0.047 | 0.19 | 64 | 0.016 | 0.094 | | 0.5 | | 55 | | |  |
| 139 | Female | 28 | Lhasa | Lhasa | GU | 0.016 | 0.023 | 1 | 0.016 | 0.064 | | 2 | | 90 | | |  |
| 140 | Female | 52 | Lhasa | Lhasa | CG | 0.016 | 0.094 | 256 | 0.094 | 0.064 | | 2 | | 85 | | |  |
| 141 | Female | 58 | Lhasa | Lhasa | CG | 0.016 | 0.047 | 256 | 0.064 | 0.047 | | 1.5 | | 86 | | |  |
| 142 | Female | 43 | Lhasa | Lhasa | CG | 4 | 0.064 | 256 | 0.064 | 0.064 | | 3 | | 80 | | |  |
| 143 | Female | 48 | Lhasa | Lhasa | CG+DU | 0.016 | 0.125 | 8 | 0.5 | 0.125 | | 6 | | 55 | | |  |
| 144 | Female | 42 | Lhasa | Lhasa | CG | 6 | 0.094 | 256 | 0.19 | 0.047 | | 0.75 | | 61 | | |  |
| 145 | Female | 42 | Lhasa | Lhasa | CG | 256 | 32 | 3 | 0.047 | 0.047 | | 4 | | 63 | | |  |
| 146 | Male | 56 | Undetermined | Undetermined | CG | 24 | 0.094 | 48 | 0.016 | 0.064 | | 4 | | 70 | | |  |
| 147 | Male | 51 | Lhasa | Lhasa | CG | 256 | 0.064 | 4 | 0.016 | 0.047 | | 1.5 | | 75 | | |  |
| 148 | Male | 53 | Lhasa | Lhasa | CG+GU | 0.016 | 0.094 | 3 | 0.047 | 0.094 | | 6 | | 65 | | |  |
| 149 | Female | 53 | Lhasa | Lhasa | GU | 0.016 | 0.032 | 96 | 0.016 | 0.016 | | 0.19 | | 70 | | |  |
| 150 | Male | 47 | Lhasa | Lhasa | CD | 0.016 | 0.032 | 32 | 0.016 | 0.032 | | 0.5 | | 80 | | |  |
| 151 | Female | 48 | Lhasa | Lhasa | CG | 0.016 | 32 | 256 | 0.064 | 0.064 | | 0.75 | | 70 | | |  |
| 152 | Female | 34 | Lhasa | Lhasa | CG | 0.016 | 0.125 | 256 | 0.032 | 0.064 | | 6 | | 55 | | |  |
| 153 | Female | 50 | Lhasa | Lhasa | CG+GU | 48 | 32 | 256 | 0.5 | 0.19 | | 6 | | 61 | | |  |

CLR, clarithromycin; LEV, levofloxacin; MTZ, metronidazole; AML, amoxicillin; TET, tetracycline; RIF, rifampicin; FZD, furazolidone; CD, chronic duodenitis; CG, chronic gastritis; DU, duodenal ulcer; GU, gastric ulcer; RE, reflux esophagitis; Ca, gastric cancer; mm, millimeter.
